# Challenges in Development of Nanoparticle-Based Therapeutics

**DOI:** 10.1208/s12248-012-9339-4

**Published:** 2012-03-10

**Authors:** Neil Desai

**Affiliations:** Strategic Platforms, Abraxis BioScience, A Wholly Owned Subsidiary of Celgene Corporation, 11755 Wilshire Blvd., Suite 2300, Los Angeles, California 90025 USA

**Keywords:** biodistribution, immune toxicity, *nab*-paclitaxel, nanoparticle, pharmacokinetics

## Abstract

In recent years, nanotechnology has been increasingly applied to the area of drug development. Nanoparticle-based therapeutics can confer the ability to overcome biological barriers, effectively deliver hydrophobic drugs and biologics, and preferentially target sites of disease. However, despite these potential advantages, only a relatively small number of nanoparticle-based medicines have been approved for clinical use, with numerous challenges and hurdles at different stages of development. The complexity of nanoparticles as multi-component three dimensional constructs requires careful design and engineering, detailed orthogonal analysis methods, and reproducible scale-up and manufacturing process to achieve a consistent product with the intended physicochemical characteristics, biological behaviors, and pharmacological profiles. The safety and efficacy of nanomedicines can be influenced by minor variations in multiple parameters and need to be carefully examined in preclinical and clinical studies, particularly in context of the biodistribution, targeting to intended sites, and potential immune toxicities. Overall, nanomedicines may present additional development and regulatory considerations compared with conventional medicines, and while there is generally a lack of regulatory standards in the examination of nanoparticle-based medicines as a unique category of therapeutic agents, efforts are being made in this direction. This review summarizes challenges likely to be encountered during the development and approval of nanoparticle-based therapeutics, and discusses potential strategies for drug developers and regulatory agencies to accelerate the growth of this important field.

## INTRODUCTION

Over the last quarter century, the field of nanotechnology has witnessed tremendous growth and advances. The area of nanoparticle-based medicine receives particular attention as it holds the promise to revolutionize medical treatment with more potent, less toxic, and smart therapeutics that could home into disease areas like the elusive magic bullet. With substantial efforts by both academia and the biopharmaceutical industry, a few nanomedicines have been successfully developed and approved for clinical use. However, there is no doubt that the field of nanomedicine is still at its early stage, with success stories few and far apart. Most nanomedicine research, while exciting and technically advanced, is in early stages of development and is only slowly being translated into clinical trials and medical practice. This review seeks to illustrate the numerous challenges that are encountered during the development of nanoparticle-based therapeutics using the commercially available protein-bound nanoparticle formulation of paclitaxel (Abraxane®, Celgene Corporation) as an example where relevant. The article also adds to the current debate about the future regulatory requirements for the approval of nanomedicines, and the challenges to be expected in the development and approval of generic equivalents of these products.

## RATIONALE FOR THE DEVELOPMENT OF NANOMEDICINES

Nanoparticles often have unique physical and chemical properties at the cellular, atomic, and molecular levels not usually seen with the bulk material (1), partially due to their high surface to volume ratio. In addition, the ability to create three-dimensional multi-component structures of nanoparticles also allows a great degree of flexibility to design drug delivery systems that may fulfill several desired properties such as the ability to overcome biological barriers, the ability to deliver hydrophobic, poorly water-soluble molecules, and the potential ability to selectively target these nanomedicines to a preferred site in the body.

### Biological Barriers to Drug Delivery

Multiple biological barriers exist for drugs to successfully reach their intended disease sites. Oral drugs need to have high stability in the gastrointestinal tract and the ability to penetrate intestinal epithelium to achieve high systemic bioavailability (2). Similarly, skin, nasal, and pulmonary drug delivery requires efficient transport of drugs across the epithelium. While most new drug development for small molecules is focused on oral delivery, with drug chemistry directed towards good oral absorption, intravenous (IV) administration remains the most direct and efficient route to deliver drugs that comprise peptides, proteins, large molecules, and polynucleotides.

Drugs in circulation still need to overcome several barriers to reach their targets. The blood–brain barrier (BBB) restricts the diffusion of large or hydrophilic molecules into the cerebrospinal fluid and is a major obstacle for treatment of most CNS and brain disorders. Multiple nanoparticle-based strategies, including liposome, nanosphere, and cationic albumin nanoparticles, are under development to deliver drugs across the BBB (3).

Overcoming the difficulty of delivering therapeutic agents into solid tumors presents another major delivery challenge. The tumor vasculature is highly heterogeneous in distribution and more permeable in some places, however, large areas of tumors may be poorly perfused (4). Impaired lymphatic drainage in tumors contributes to increased interstitial fluid pressure (IFP) (5) adding another barrier to delivery. The elevated IFP has been described to be one of the main factors contributing to limited extravasation and transvascular transport of macromolecules despite the leaky tumor microvasculature, and inhibits the transport of molecules in tumor interstitial space (6). High tumor cell density and dense tumor stroma can further hamper the movement of drugs within tumors (4). In particular, the presence of collagen in tumor extracellular matrix is a major factor limiting drug penetration in the tumor interstitium, with pancreatic cancer being a primary example (7). Although tumors pose these barriers for drug delivery, the pathology and natural biological mechanisms of tumors, yet unexplored, leave great opportunities for targeting by nanomedicines.

**Fig. 1:**
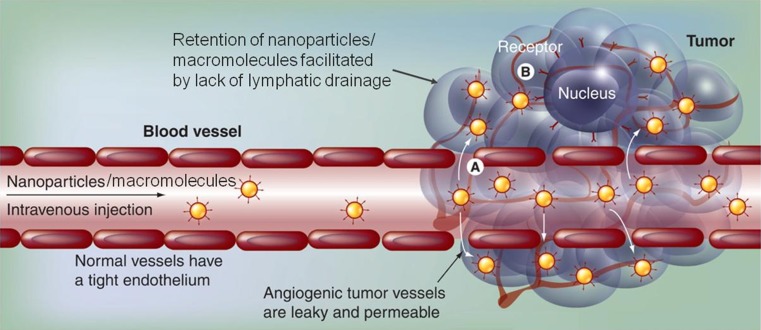
Enhanced drug delivery to solid tumors using nanoparticles or macromolecules (8). *A* Passive delivery (EPR). After IV injection, nanoparticles accumulate in tumors through leaky and permeable tumor vasculature and impaired lymphatic system. *B* EPR + targeted delivery. “Targeted” nanoparticles or macromolecules bind to cancer cell receptors resulting in potentially improved drug delivery. (Adapted from Nanomedicine, June 2010, vol. 5, no. 4, pp 597–615 with permission of Future Medicine Ltd.)

**Fig. 2:**
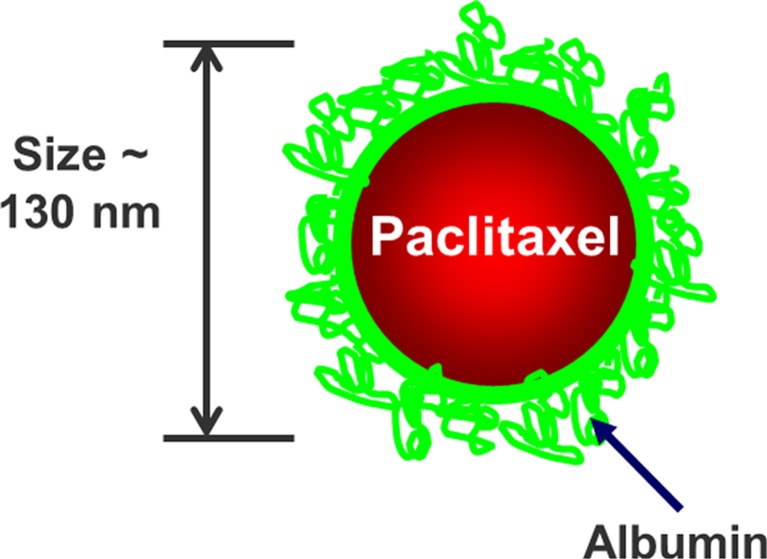
Abraxane (*nab*-paclitaxel) schematic

**Fig. 3:**
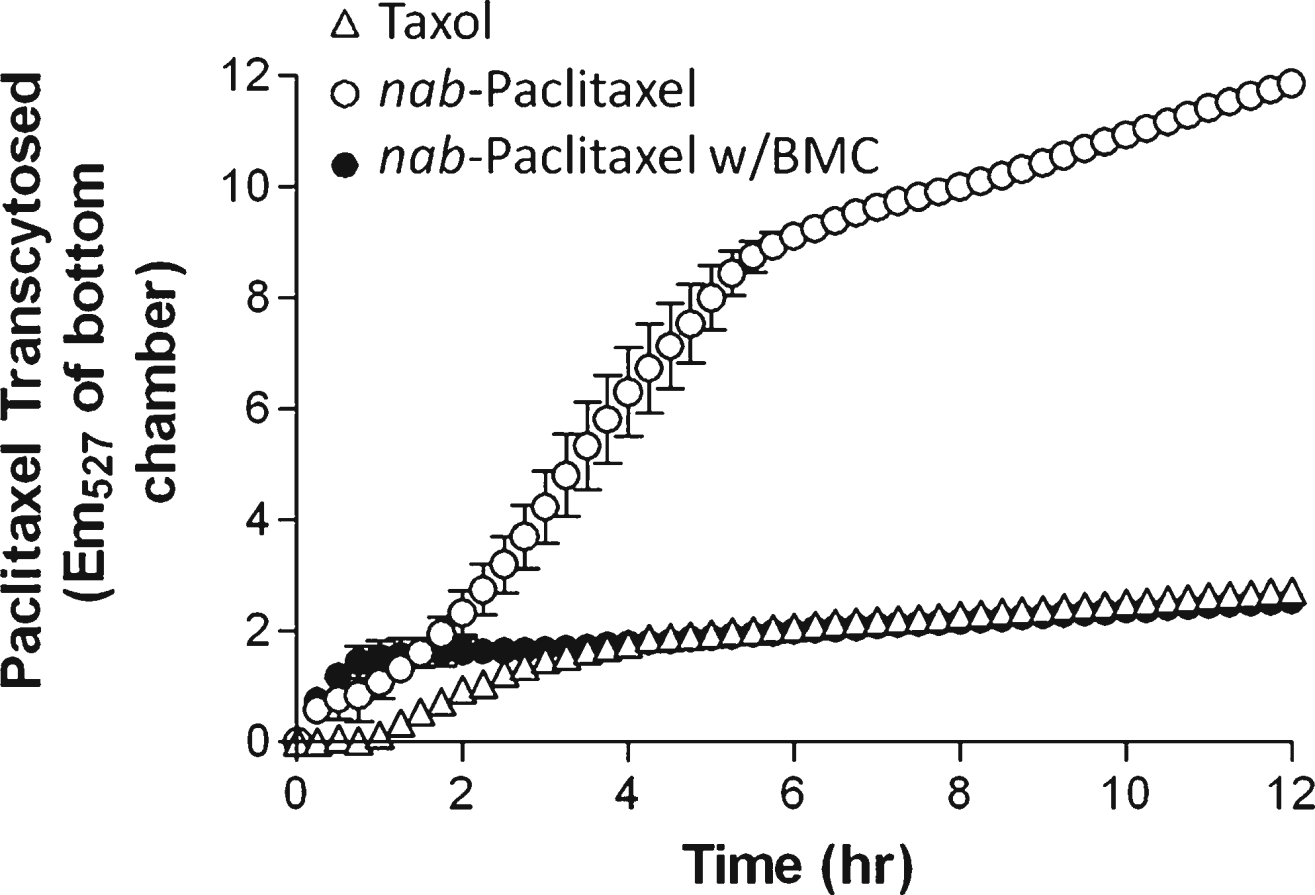
*nab*-paclitaxel (ABI-007) transcytosis across endothelial cells as an assay demonstrating “biological function” or transport due to albumin (22). Transcytosis of paclitaxel across human umbilical vascular endothelial cells was significantly enhanced in *nab*-paclitaxel compared with Cremophor-based paclitaxel (Taxol). The enhanced transcytosis of *nab*-paclitaxel was inhibited by β-methyl cyclodextrin (*BMC*), a known inhibitor of the gp60/caveolar transport

The high permeability of tumor vasculature and the lack of proper lymphatic drainage result in the so called enhanced permeability and retention effect (EPR) in the tumor microenvironment (Fig. 1), which has been suggested to improve tumor drug delivery for nanoparticles and macromolecules that have the ability to circulate with a long half life (8,9). While nanomedicines may take advantage of this passive process, the utilization of an active biological transport process may further improve drug delivery. For example, Abraxane® (paclitaxel protein-bound particles for injectable suspension (albumin bound), also known as *nab*®-paclitaxel or ABI-007, Abraxis BioScience/Celgene; Fig. 2) can achieve rapid tumor penetration and enhanced accumulation by utilizing the albumin transport pathways, including gp60 albumin receptor and caveolae-mediated endothelial transcytosis (Fig. 3) across endothelium of the blood-tumor barrier and potential interaction with albumin binding proteins (e.g., secreted protein, acidic and rich in cysteine (SPARC)) in the tumor interstitium (10).

### Delivery of Hydrophobic Drugs

The efficient and safe delivery of hydrophobic therapeutic compounds remains a serious hurdle for the pharmaceutical industry. The formulation of many hydrophobic drugs requires toxic solvents and surfactants such as Cremophor and Tween, which often impair drug distribution and are associated with severe side effects. For example, Taxol®, the conventional formulation of the hydrophobic drug paclitaxel (Bristol-Myers Squibb Co), contains a high concentration of Cremophor-EL® (BASF, Ludwigshafen, Germany), a solvent associated with significant toxicities including potentially lethal hypersensitivity, anaphylactic reactions, and prolonged peripheral neuropathy (11,12). Cremophor can also sequester paclitaxel in micelles, which prolongs the systemic exposure and increases drug toxicity (13). Polysorbate, another commonly used solvent for hydrophobic drugs, can also induce hypersensitivity reactions (14).

Nanomedicines which do not require the use of toxic solvents offer clear advantages over their conventional counterparts. The first protein nanotechnology-based chemotherapeutic approved, *nab*-paclitaxel, is a Cremophor-free, albumin-bound nanoparticle formulation of paclitaxel with a mean particle size of approximately 130 nm. By eliminating the Cremophor EL/ethanol vehicle used in Taxol and the associated toxicities and incorporating nanotechnology, *nab*-paclitaxel confers the ability to achieve a 50% higher maximum tolerated dose (300 *vs*. 175 mg/m^2^ for solvent-based paclitaxel, every 3 weeks), a shorter infusion time (30 min *vs*. 3 to 24 h with solvent-based paclitaxel) without the need for premedication to prevent solvent-related hypersensitivity reactions. *nab*-paclitaxel also has linear pharmacokinetics (PK) due to the avoidance of micelle entrapment which occurs with solvent-based paclitaxel. In addition, *nab*-paclitaxel can use standard tubing and IV bags (10).

### Desire for Targeting

Targeting increases efficacy, decreases side effects, and reduces systemic drug exposures. The organized structures of nanoparticles allow for incorporation of various targeting moieties to enhance drug delivery to the target sites, reduce off-target organ toxicities, and facilitate cellular uptake of therapeutic agents (15). The distinctive biology and pathology of tumors make effective delivery a high priority for antitumor therapeutics, as well as present an endless list of potential candidates for targeting (Fig. 1). Most active targeting moieties of nanoparticles are biologics, including ligands for receptors, peptides, proteins, and antibodies. Studies have shown that nanoparticles carrying ligands or monoclonal antibodies targeted to surface receptors overexpressed by cancer cells, such as the transferrin receptor, the folate receptor and EGFR, can increase cellular internalization of the agents through endocytosis and improve the efficacy of systemic anticancer therapy (16,17). In addition, nanoparticles incorporating cell-penetrating peptides and protein-transduction domains, such as oligo-arginine and TAT, enable the uptake of agents that otherwise cannot effectively enter cancer cells (18). Further, some nanomedicines are designed to target tumor endothelial cells. For example, cyclic and linear derivatives of the oligopeptides RGD, through binding to the integrins α2β3, αvb3 and α5β1, can direct liposomes and other nanoparticles carrying vascular targeting and cytotoxic agents to endothelium and tumor cells, thus achieving significant antitumor activity (19–21).

## CHALLENGES IN NANOMEDICINE FORMULATION, CHARACTERIZATION AND MANUFACTURING

Nanomedicines are likely to be three-dimensional constructs of multiple components with preferred spatial arrangements for their functions. As a result, subtle changes in process or composition can adversely affect the complex superposition of the components with negative consequences. A thorough understanding of the components through detailed physicochemical characterization as well as functional tests may be essential in order to support highly reproducible manufacturing processes for nanomedicines.

Multiple hurdles exist before a nanomedicine can reach the clinic, starting with detailed characterization and the successful manufacture of these complex constructs. Other than the standard criteria for acceptable safety and efficacy, and desirable pharmaceutical characteristics (e.g., stability, ease of administration, etc.) that are applicable to most drugs, the ideal nanoparticle system or nanomedicine to be utilized for therapeutic purpose may embody the following features:

- Detailed understanding of critical components and their interactions
- Identification of key characteristics and their relation to performance
- Ability to replicate key characteristics under manufacturing conditions
- Easy to produce in a sterile form
- Ability to target or accumulate in the desired site of action by overcoming the restrictive biological barriers
- Good in-use stability, easy to store and to administer

### Nanoparticle Components and Characteristics

The versatility of nanoparticles allows for a potentially wide variety of choices in the payload, nanoparticle composition, and targeting moieties. The *in vitro* and *in vivo* properties of nanoparticles depend on a number of key physicochemical characteristics, including size and size distribution, surface morphology, surface chemistry, surface charge, surface adhesion, steric stabilization, drug loading efficiency, drug release kinetics, and hemodynamic properties of the nanoparticles.

Nanoparticles have been adapted to deliver different kinds of therapeutic agents, including small molecule drugs, peptides, proteins, oligo- and polynucleotides, and genes. The nanoparticles used in drug delivery include liposomes, polymers, proteins, micelles, dendrimers, quantum dots, nanoshells, nanocrystals, gold nanoparticles, paramagnetic nanoparticles, and carbon nanotubes (23). Each of these systems has widely varying architecture and attempting to generalize key physicochemical characteristics between the different approaches may be futile. Therefore, it is beneficial that in each particular system, key characteristics and critical components that may dictate the performance of the nanoparticle system or particular nanomedicine be defined and understood by the innovators.

Particle size and size distribution is one of the most widely accepted defining characteristic of nanoparticle-based medicines because size can significantly impact the PK, biodistribution, and safety. After administration, small nanoparticles with size smaller than 20–30 nm are rapidly cleared by renal excretion, while particles 200 nm or greater in size are more efficiently taken up by the mononuclear phagocytic system (MPS; also known as reticuloendothelial system), with cells in the liver, spleen, and bone marrow (24). Previous reports have shown that nanoparticles of 150–300 nm locate mainly in the liver and spleen (25), and colloids of sizes 200 to 400 nm undergo rapid hepatic clearance (26). It has been well described that tumor blood vessels are leaky with fenestrations ranging between 0.2 and 1.2 μm, therefore nanoparticles with size below 200 nm can take advantage of the EPR effect for enhanced drug accumulation in tumors (27–29). Particles will always exist in a range of sizes, therefore, size distribution must also be taken into account when designing a nanomedicine. Considering a normal size distribution, for the vast majority of particles to be below 200 nm in size, the mean nanoparticle may need to be well below 200 nm to confer the full “benefits” of a nanomedicine. Therefore, the nanoparticle size and size distribution need to be carefully controlled during the small-scale preparation and in particular during a larger-scale manufacturing process.

Nanoparticle surface properties are also critical determinants for nanoparticle behaviors and interaction with proteins and cells (30). A multitude of surface characteristics (charge, hydrophobicity, functional groups, etc.) play an important role in nanoparticle stability and the opsonization process (24,31,32). Upon entering circulation, colloidal nanoparticles are coated with various blood components (such as albumin, fibrinogen, IgG, and apolipoproteins) in the opsonization process, which activates the complement pathway and targets the particles for clearance by macrophages (26,33). Complement activation by nanoparticles is also sensitive to surface polymer conformation (34). As an example, macrophages can directly recognize nanoparticles via the particle surface. Particles with hydrophilic surfaces can become more hydrophobic in circulation by the adsorption of IgG, whereas hydrophobic particles can be directly taken up by macrophages without opsonization (26). Poly(ethylene glycol) (PEG) and other polymers can provide a hydrophilic surface and protect nanoparticles from opsonization and immune recognition (35).

The choice of a suitable targeting moiety for the intended disease could play important roles in enhancing efficacy and reducing side effects for a nanomedicine. Active and biological targeting moieties can alter PK, biodistribution, and cellular uptake. For instance, alteration of the density of surface ligands can potentially elicit complement activation and other immune responses, further complicating the composition of multifunctional and multicomponent nanoparticles (36–38).

Identifying the “right” nanoparticle parameters for the intended indication is crucial. Moving in one direction could solve a particular problem but may often lead to another issue. In this respect the example of Doxil® and Myocet® is quite illustrative. Conventional liposomes termed as “non-stealth” have high affinity for the MPS and are rapidly removed from circulation. In contrast, stealth liposomes with a pegylated coating can significantly decrease their uptake by macrophages. PEGylated liposomal doxorubicin (Doxil®) demonstrates a prolonged half life, increased tumor drug concentration, better antitumor efficacy, and fewer side effects than conventional doxorubicin (39). While the PEG coating of Doxil limits cardiotoxicity, it also results in higher concentrations in the skin resulting in a side effect called palmar plantar erythrodysesthesia, more commonly known as hand–foot syndrome (39). In addition, PEGylated liposome infusion has shown idiosyncratic non-IgE-mediated signs of hypersensitivity in humans, which can be reduced by slowing the rate of infusion or by premedication (40). In contrast, Myocet® (Enzon Pharmaceuticals) is a non-pegylated liposomal doxorubicin approved in Europe and Canada for treatment of metastatic breast cancer in combination with cyclophosphamide (41). The difference in compositions allows Myocet to have reduced cardiotoxicity but no hand-foot syndrome (42).

Currently, there are no good *in vivo* models to predict the diverse behaviors of the many types of nanoparticles under investigation, so the development of nanoparticles with desirable properties has to rely on empirical evidence and extensive preclinical animal testing.

### Analysis and Characterization of Nanoparticle Formulations

Identifying the appropriate analytical tests to fully characterize nanomedicines, whether physical, chemical or biological, may be one of the more challenging aspects of nanomedicine development both from a technical as well as regulatory perspective. Due to the complex nature of nanomedicines compared with standard pharmaceuticals, a more sophisticated level of testing should be required to fully characterize a nano product. Because each component of a nanomedicine serves a specific function, it would be essential to be able to quantify each component and also evaluate the relationships and interactions between these components in aspects that include both stoichiometry and their spatial orientation. Multiple orthogonal characterization techniques are essential to ensure that the nanomedicines have all the desired properties for the intended therapeutic purpose.

While the standard analytical tests such as quantification of active and inactive ingredients, impurities, etc. in pharmaceutical products still apply, various additional techniques are employed specifically for the characterization of nanoparticle physicochemical properties. These tests involve a broad range of methods, including visualization of nanoparticles by microscopy (atomic force microscopy, transmission electron microscopy (TEM), and scanning electron microscopy); measurement of particle size and size distribution with light scattering (static and dynamic), analytical ultracentrifugation, capillary electrophoresis, and field flow fractionation (25); analysis of surface charge or zeta potential; and examination of surface chemistry by X-ray photoelectron spectroscopy or Fourier transform infrared spectroscopy (43). The crystalline state of drugs encapsulated in the nanoparticles can be assessed by X-ray diffraction and differential scanning calorimetry.

More innovative testing methods are constantly being developed and applied to the analysis of nanoparticles. The key parameters, as well as the overall stability of nanoparticles, should be tested with nanoparticles in solid form, in suspension, and in biological medium, and under accelerated conditions such as higher temperature to ensure the robust performance of nanoparticles. However, the above tests may not be able to functionally differentiate between an “active” formulation and one that is “inactive” or “less active.”

As an example, consider a nanoparticle carrying a payload of an active drug and having at its surface moieties that allow stealth features, such as PEG and moieties that may allow targeting including peptides, nucleic acids, proteins, or antibody fragments that bind certain receptors. Quantitative techniques to analyze the individual components, while essential, will be missing key information about the distribution of these moieties on the surface of the particles or whether indeed these moieties are at the surface or buried within the structure of the nanoparticle. The spatial distribution of these moieties is critical for the intended function of the particular nanoparticle. A different series of tests may be required to determine these aspects; such tests are likely to be non-conventional in pharmaceutical use, including surface analysis, as well as biological function tests to assure that the manufacturing process produces nanoparticles that are active in cellular uptake, transcytosis (Fig. 3), or binding to appropriate biological materials. These “structure-function” tests are essential at least up to the point where one can validate a highly reproducible manufacturing process. Bioassays to confirm activity of the product are often used when testing biologic drugs.

Several proposed nanotherapeutics have complex components such as proteins or nucleic acids forming an integral part of the nanomedicine (44,45), which may be sensitive to the manufacturing process conditions and in some cases undergo change in composition as a result of the manufacturing. These components may not be the “active” pharmaceutical ingredient in the nanomedicine, but their presence may serve a role in targeting specific cells/biological pathways or distribution of the active ingredient in the body. These components cannot be considered as inactive excipients due to their important role in efficacy or safety of the product, and should be fully characterized by appropriate analytical tests.

In addition to finished product tests, it is useful to institute appropriate in-process tests during early manufacturing development that can provide essential information during the development of a robust manufacturing process. These are tests that can be conducted with relatively quick turnaround and are designed to give an early read into how varying process conditions can affect the nanomedicine composition at intermediate stages of the process.

In summary, due to the complex nature of nanomedicines, it is reasonable to expect that the level of analytical characterization, testing and release required to adequately understand and define the physicochemical or biological nature of these products is more sophisticated and burdensome than for standard pharmaceutical products. The better one can understand these products in early stages of development, the more likely it is that a successful reproducible manufacturing process will be achieved.

### Scale-up and Manufacturing

The successful scale-up and manufacturing of a nanomedicine present unique challenges in pharmaceutical development. Conventional pharmaceutical manufacturing does not typically create three dimensional multicomponent systems in the nanometer scale and as such this requirement presents a series of obstacles for the scale-up of nanomedicines.

Since most nanoparticles are complex multicomponent products with specific arrangement of components, a full understanding of the components and their interactions is essential to defining the key characteristics of the product. Identifying these characteristics early in development in turn, greatly helps define larger scale manufacturing in order to establish critical process steps and analytical criteria that ensure reproducibility of the product.

The methods of nanoparticle preparation can be broadly categorized into “top–down” and “bottom–up” approaches. Top–down approaches seek to create smaller entities from larger ones, such as grinding of particles using the milling technique. In contrast, bottom–up approaches arrange smaller components into more complex assemblies, which often involve polymerization of monomers or molecular self-assembly to cause single-molecule components to automatically arrange themselves into a useful conformation (43).

The nanoparticle formulation process often involves the use of organic solvents, high-speed homogenization, sonication, milling, emulsification, crosslinking, evaporation of organic solvents, centrifugation, filtration, and lyophilization. During early development, at the lab- or small scale, it is useful to consider what approach may be useful if the product were to be scaled up. Identification of important process conditions is critical to achieve key attributes and functions. These conditions may involve the ratio of polymers, drugs, targeting moieties, the type of organic solvent, and emulsifier/stabilizer/crosslinker, the oil-to-water phase ratio, mixing, temperature, pressure, and the pH (43). Depending on conditions, the process may lead to altered chemical structure of the active and the other components and substantial amount of impurities. For macromolecules, particularly biologics, it may result in changes in chemical structure and conformation, denaturation, crosslinking, coagulation, and degradation. Importantly, nanoparticles are not simple additions and mixtures of individual components. Rather, they are integral and highly structured compositions. The structural integrity and physicochemical properties of intact nanoparticles must be preserved throughout the formulation process to the finished product.

A critical consideration is that the formulation process must be robust to ensure high reproducibility, and be streamlined to allow for the ease of scale-up production. The manufacture of nanoparticles often requires multiple process steps involving multi-component systems. Although small-scale processes may achieve reproducibility with well characterized components, once beyond the early prototype, the reproducibility and consistency of the constructs remain a constant challenge for the scale-up and manufacturing process. The manufacturing plan needs to define acceptable limits for key nanoparticle attributes and identify process conditions that are critical to achieve these key attributes and functions. These critical conditions must be identified at the small scale through extensive experimentation to gain a full understanding of how process conditions can impact the product both from a physicochemical and biological perspective. This necessitates that the physicochemical and biological tests be sensitive enough to identify discrepancies in the product that could affect performance (see “Analysis and Characterization of Nanoparticle Formulations”). In multistep processes, in-process testing for critical parameters with a rapid and reliable analytical method is often very informative about how well the process is controlled. Building up a database of information with suitably targeted in-process tests may be vital to ensure success at the manufacturing scale.

Liposomes provide a good example to illustrate issues of stability, scale-up and importance of critical process parameters. Differing preparation methods can create liposomes with multilamellar vesicles, small unilamellar vesicles, or large unilamellar vesicles. Each of these systems may be unstable due to high-surface free energy and the tendency to aggregate. The use of amphipathic PEG on the surface can inhibit liposome aggregation by reducing interfacial free energy with water and acting as a steric barrier, therefore improving stability in addition to extending *in vivo* circulation half-life of liposomes (46–48). The physical stability of liposome drug products is determined by the integrity, size distribution, and composition of liposome vesicles. The FDA has highlighted the importance and challenges in maintaining a close control over the manufacturing process in a draft guidance for liposome drug products stating that “liposome drug products are sensitive to changes in the manufacturing conditions, including changes in scale. This should be considered during the development process, and critical manufacturing parameters (e.g., scale, shear force, and temperature) should be identified and evaluated” (49).

Nanomedicines that are to be utilized by a route of administration that requires a sterile product will face particular challenges dependent on their particle size and composition. Nanomaterials are known to be at increased risk for being damaged by sterilization techniques such as gamma irradiation or autoclaving, especially when biological materials are involved (50–52). If the structure of the particles is flexible or malleable, such as in case of some liposomal preparations, then sterilization through conventional sterile filters may not be problematic especially if the starting particle size is well below 220 nm (0.22 μm). In the case of rigid structures such as polymeric, silica-based, metal and other nanoparticles, sterilization by filtration may be the only option, however, wide particle distributions and particle sizes closer to 220 nm can result in tremendous difficulty in filtration due to the nominal pore size of standard filtration membranes. If the mean particle size is not well below 220 nm, then substantial amounts of the active ingredient could be lost on filtration. While aseptic manufacturing is always an option, this can be quite complicated, in particular for a multiple step process that involves handling and transfer of materials in a “sterile” environment.

One of the other challenges is related to the reproducibility of *in situ* preparation of nanomedicines. Some nanomedicines have utilized the concept of “self-assembly” and “*in situ*” preparation where two or more components as intermediates ready to use at the “bedside” are brought together under appropriate conditions to create structures or complexes as the final finished product for human use (53–55). This approach, by manufacturing individual components, circumvents the otherwise complex manufacturing steps of creating a finished nanomedicine as a stable pharmaceutical product and may significantly reduce the cost of manufacturing and forego the complex development work it would involve. Despite these advantages, the *in situ* strategy raises certain questions. If the three-dimensional complex structure of nanomedicines is critical to their function, then can one rely on the vagaries of the individuals in the hospitals or doctor’s offices to create a “reproducible” finished product for administration in these different settings? Are these individuals performing a critical part of the manufacturing step essentially at the bedside of the patient? Would the *in situ* final products be subject to the same stringent standards that may be applied to a shelf stable nanomedicine product to ensure reproducibility and control of the manufacturing process? Or, should there be qualifying release tests for *in situ* products as is required for a shelf stable product when released for distribution? Some of these issues need careful consideration as policies and guidelines evolve for nanomedicine products.

An additional issue for the manufacture of nanoparticles is environmental safety. The handling of dry materials in the nanometer size scale demands special caution as airborne nanoparticles distribute as aerosols. Lung deposition of such nanoparticles can lead to pulmonary toxicities (56,57). During dosing solution preparation, aerosolization of solutions needs to be avoided to prevent unintended exposure. Some nanoparticles are capable of penetrating the skin barrier, making dermal exposure a potential risk so adequate protection of personnel is essential (56). In this respect, nanoparticles that are created entirely within a liquid environment may have significantly lower environmental impact, presumably no different from standard manufacturing of liquid pharmaceutical products.

### Challenges in the Development of *nab*-paclitaxel

The development of *nab*-paclitaxel illustrates well the challenges in formulation, manufacturing, and testing of a nanoparticle with appropriate physicochemical properties. As the first approved protein-based nanomedicine, *nab*-paclitaxel underwent extensive preliminary testing in small scale formulation. A wide range of conditions were investigated for the manufacturing process, as well as protein from different sources with variations in quality and purity. Differing conditions of preparation often resulted in suboptimal preparations, a challenge that could be overcome only with large amounts of trial and error. These hurdles for successful scale-up production of *nab*-paclitaxel were further illustrated by unsuccessful attempts in the marketplace to copy the *nab*-paclitaxel formulation. This aspect is discussed in the “Regulatory Challenges to Nanomedicine Development” section of this review. A large number (several hundred) of optimization runs/batches were conducted to define components and compositions of the nanoparticle and to develop a robust process that performs with consistency and reproducibility for scale-up manufacture.

**Fig. 4:**
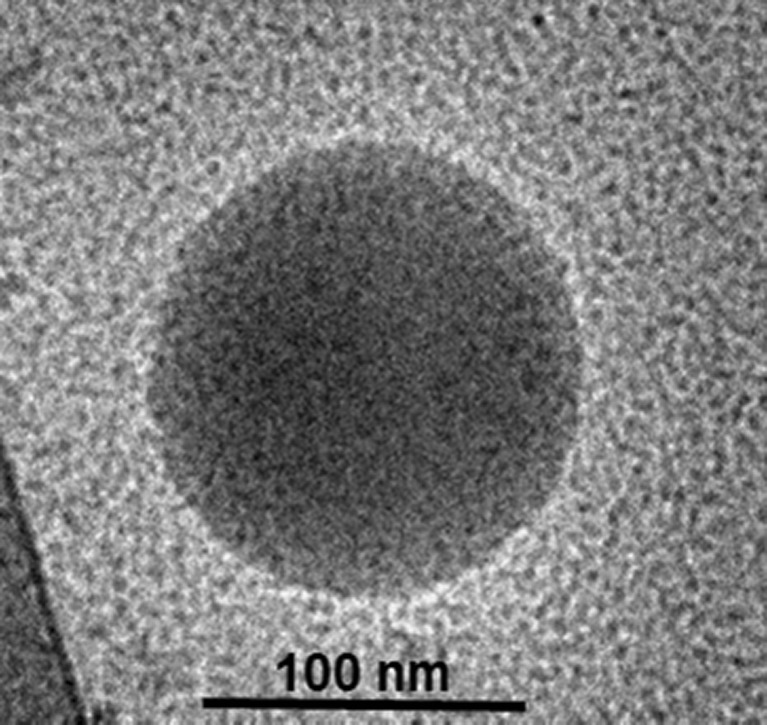
Cryo TEM image of *nab*-paclitaxel showing spherical nanoparticle

As a result, the *nab*-paclitaxel nanoparticles displayed many features desirable for an injectable nanomedicine. The size distribution of *nab*-paclitaxel nanoparticles in solution was narrow with a mean particle size of 130 nm as determined by dynamic laser light scattering (58). TEM and cryo-TEM images revealed that the nanoparticles were spherical with sizes well below 200 nm (Fig. 4). The albumin surface of *nab*-paclitaxel nanoparticles has a high negative zeta potential which along with steric stabilization by albumin, prevents agglomeration and provides the nanoparticles with good stability in suspension. X-ray powder diffraction revealed that paclitaxel within the nanoparticles is non-crystalline (amorphous), making the drug readily bioavailable without the time lag needed to dissolve crystalline paclitaxel as is well known for nanocrystals (59). *nab*-paclitaxel comprises nanoparticles of drug coated with a layer of albumin crosslinked to a specific level, with paclitaxel non-covalently bound to albumin via hydrophobic interaction, which allows for high bioavailability and rapid tissue distribution. This is in contrast with most of other albumin-based nanoparticles reported in literature (60–62), which involve the addition of glutaraldehyde or other crosslinking agents during nanoparticle formation and require enzymatic degradation of albumin for drug release *in vivo*.

In summary, the careful selection of key components, identification of key characteristics and understanding of critical manufacturing steps are determinants of whether the nanoparticles will have the desired pharmacodynamics, PK, and safety profiles to achieve the intended therapeutic effects. Multiple orthogonal analysis methods are required for appropriate in-process quality controls and tests for finished products. Deviations from key nanoparticle parameters and processes could have serious negative impacts on safety and efficacy of a nanomedicine.

## PHARMACOLOGY AND SAFETY CHALLENGES OF NANOMEDICINES

Because the pharmacological and safety profiles of nanomedicines are influenced by the cumulative contribution of physicochemical characteristics, subtle changes in composition arising from small deviations in the manufacturing process could result in substantial changes in pharmacology and toxicity of nanomedicines.

### Pharmacology Issues Related to Nanomedicines

It is essential for a successful nanomedicine to achieve the desired pharmacological profile and PK profile suitable for the intended indication. However, several challenges are associated with trying to apply the standard criteria of small molecule PK to the PK of nanomedicines.

Usually, only a small fraction of the administered drug reaches its intended location and because of this, the standard approach of determining PK in the blood or plasma as the sole measure of *in vivo* behavior of nanoparticles may be inherently flawed. While small molecule drugs may diffuse more readily through “biological barriers” and hence a blood level may be somewhat in equilibrium and related to achievable target tissue levels, applying this logic for larger macromolecular complexes and nanomedicines cannot be assumed to be correct. It is well recognized that small or subtle compositional differences can affect the biodistribution of nanoparticles or nanomedicines (1,63,64). With the wide variety of potential nanomedicines on the horizon, it would be highly unlikely that standard pharmacological approaches would be appropriate to characterize their behavior. Accordingly, pharmacology at the site of action may be more appropriate to define if a particular nanomedicine approach achieves reliable target tissue concentrations.

The PK and biodistribution of the active drug within the nanomedicine may be affected by several factors. Subtle compositional aspects, size, shapes, and physicochemical properties of nanoparticles can result in altered PK and greater PK variations compared with conventional small molecule approaches. Nanomedicines may allow for novel routes of delivery including oral, pulmonary, and dermal administration, which requires high bioavailability through the biological barriers. The PK of both the nanomedicine as a whole as compared with just the “free” drug may be highly relevant.

The clearance of nanoparticles *in vivo* is a complicated process controlled by numerous characteristics such as particle size, surface properties and possibly other compositional characteristics that are not fully understood at this time. Smaller nanoparticles are cleared by the kidney, whereas larger particles are cleared by the Kupffer cells and macrophages of MPS located predominantly in the liver and spleen (24). The nanoparticle clearance is facilitated by the opsonization of blood components and complement proteins on the particle surface (31). The inhibition of opsonization and evasion of detection by macrophages with approaches such as pegylation prolong the circulation of nanoparticles in the case of liposomal doxorubicin (Doxil) (65).

The size and composition can affect the release kinetics of the active drug from the nanomedicine carrier. Whereas monodisperse nanoparticles or particles with narrow distribution are desirable for consistency, it has also been proposed that nanomedicines can be composed of a nanoparticle population of mixed sizes to intentionally introduce different rates of drug release for sustained delivery over time. Additionally, both passive targeting (e.g., clearance by the MPS in cases where the liver is the target or where longer circulation time for EPR is desired) and active targeting approaches may significantly enhance the distribution and accumulation of nanomedicine drugs at the intended target sites.

There is no uniformly effective approach to design a nanomedicine to achieve a desired PK profile. A common approach has been to try to get long circulation times with nanomedicines to take advantage of EPR effect or targeting (9). However, this approach may not always be appropriate for the desired indication, and in some cases could reduce therapeutic efficacy and unnecessarily increase systemic exposure. Alternate approaches may also have benefit as in the case of *nab*-paclitaxel. Rather than utilizing passive transport via the EPR effect for drug delivery to tumor, *nab*-paclitaxel relies on active albumin transport pathways including the gp60/caveolae-mediated albumin transcytosis across tumor blood vessel endothelium and potential association with tumoral SPARC to achieve enhanced drug accumulation in tumors (10). Desai *et al*. have shown that compared with Cremophor-based paclitaxel (Taxol), *nab*-paclitaxel formulation increased the endothelial binding of paclitaxel by 9.9-fold (*P* < 0.0001) and the transport of paclitaxel across microvessel endothelial cell monolayers by 4.2-fold (*P* < 0.0001) (16). Compared to non-linear PK of conventional Cremophor-based paclitaxel (Taxol®), *nab*-paclitaxel exhibits a linear PK profile with faster clearance and increased volume of distribution, which is contrary to the typical approach for nanoparticle-based medicines (66). Caveolar transport utilizing *nab* formulations has been demonstrated *in vivo* in a live rat model of transpulmonary absorption. The epithelial and endothelial lining of alveolae is known to be rich in gp60 and caveolar transport (67–69). Intratracheal administration of *nab*-paclitaxel showed a rapid uptake into the blood stream with a plasma concentration profile from pulmonary delivery of these nanoparticles essentially matching the plasma concentration for the intravenously delivered drug (70). These results suggested that the caveolar transport process was a potentially dominant pathway for *nab*-drug transport in the lung.

As discussed above, standard PK may not be sufficient for evaluating nanoparticle-based medicines, as plasma PK is not necessarily representative of PK in tumors and disease sites, and therefore cannot predict clinical activity. The PK at site of action is more relevant and can be better correlated with therapeutic outcome. This is especially true for targeted nanomedicines. For example, despite faster clearance of *nab*-paclitaxel from circulation, intravenously administered *nab*-paclitaxel achieved 33% higher intratumoral paclitaxel concentration than equal dose of Taxol in tumor xenografts (16). In a retrospective analysis of non-small cell lung cancer patients treated with the combination of *nab*-paclitaxel and carboplatin, no correlation was observed between drug exposure based on plasma PK parameters and clinical efficacy as measured by overall response rate and progression-free survival (Abraxis BioScience, unpublished data). This could be due to the lack of correlation between tumor drug exposure and plasma PK, and potential differences in drug resistance among patients. Further, the interpatient PK variability of a liposomal CKD-602 was observed to be several-fold higher compared with a conventional formulation (64) suggesting that nanomedicines may be susceptible to higher variability in PK due to variable clearance mechanisms.

In summary, the development of nanoparticle-based medicines faces numerous pharmacological challenges. The PK profile of nanoparticle-based medicines can be influenced by variations in many different parameters and therefore, nanomedicines may require different PK approaches for different indications. Furthermore, instead of standard testing of plasma PK, physicochemical properties and drug concentrations or accumulation at the disease site may be more relevant to evaluate the reproducibility and activity of nanomedicines. In the future, these methods may also be useful for evaluating bioequivalency of nanotechnology-based products.

### Safety Challenges in Nanomedicine Development

In recent years there has been increasing attention to toxicities unique to nanoparticle-based medicines (71,72). Regulatory bodies have engaged in several public discussions on this topic and in some cases have published their findings (73). The consensus has been that each product may have its own case by case issues requiring particular investigations. In general, the standard battery of formal toxicology analyses in the preclinical setting that are conducted for any new drug should be sufficient to catch any tissue specific adverse outcome with a nanomedicine. This may be a good guiding principle, however, it should be recognized that additional testing in the preclinical setting may be required that is specific for the behavior of the particular product. As an example, in case of the materials that are persistent, not readily excreted, eliminated or metabolized, or reside in particular tissues for extended periods, it is reasonable to expect a regulatory agency to require that the consequences of the longer persistence be fully evaluated. In contrast, nanomaterials that can be proven to be rapidly eliminated from the body may not require protracted testing.

Important and unique to nanomedicines is the safety of the nanoparticulate system as a whole. International standard-setting bodies have recognized this implication and agreed that “as a minimum set of measurements—size, zeta potential (surface charge), and solubility” of nanoparticles should be used as predictors of nanoparticle toxicity (74). For example, when inhaled, nanomaterials less than 100 nm can induce pulmonary inflammation and oxidative stress (56) and disrupt distal organ functions through mechanisms including hydrophobic interactions, redox cycling, and free radical formation. Unstable nanoparticles may form large aggregates in micrometer size scale, which can be entrapped in the capillary bed of the lungs and pose a serious danger to patients. Notwithstanding these suggestions, it should be recognized that standard toxicology studies required before moving a product into the clinic will more than likely pick up any manifestation of such toxicities due to the extensive histopathology required.

### Immunological Challenges of Nanomedicines

However, one set of toxicities that cannot readily carry over from preclinical testing to humans is immunotoxicity. The immune response can be elicited by different sources. Biologics such as proteins, peptides, antibody fragments, and nucleic acids in nanoparticles can serve as antigens. Interaction of drug and carrier can result in conformation changes that increase immunogenicity. For example, immunological issues can arise from paclitaxel interacting with albumin (75). In the case of *nab*-paclitaxel, an immunological type response was observed in pigs with *nab*-paclitaxel drug but not the albumin control, supporting the observations reported by Trynda-Lemiesz *et al*. (72). The conjugation of C60 fullerene derivatives to bovine serum albumin (BSA) resulted in the generation of particle-specific antibodies and was used for immunization (76,77). Polyamidoamine dendrimers conjugated to BSA also showed increased antigenic potentials and induced dendrimer-specific antibody (78). The complex manufacturing process of nanoparticle-based medicines presents many opportunities for endotoxin contamination, which is also a source for immune response.

Nanoparticles can be antigenic themselves, with the immunogenicity of nanoparticles being affected by their size, surface characteristics, charge, hydrophobicity, and solubility. Depending on these properties, some nanoparticles can be opsonized by plasma proteins and recognized as foreign bodies, resulting in the activation of complement pathway. The complement activation can lead to rapid phagocytosis and clearance by macrophages of the MPS system in the liver and spleen, such as in the cases of superparamagnetic iron oxide nanoparticles Ferumoxtran-10 (Combidex) and ferumoxytol (Feraheme) (79). More importantly, complement activation may also provoke undesirable consequences including life-threatening allergic, anaphylactic and hypersensitivity reactions, as well as activation of humoral and cellular immune responses against the nanoparticles (72,80,81). For example, Abrams *et al*. reported that liposomal siRNA delivery vehicle LNP201 induced cytokine release typical of unregulated innate immune response, which in the most severe form can lead to the so-called “cytokine storm” (82). While PEG or other types of polymers can shield nanoparticles from immune recognition, reports have shown that administration of PEG-coated liposomes results in the formation of PEG-specific antibodies, which accelerate clearance of the PEG-liposomes and alters their PK and safety profiles (83–85). This is well recognized for liposomal doxorubicin, which requires premedication as a part of the administration regimen to ameliorate the cytokine effects and also shows a change in PK overtime due to increased clearance (86).

Nanoparticles have also been reported to increase antigenicity of weak antigens and thus serve as adjuvants. This proves to be useful in the development of nanoparticle-based vaccines but is detrimental to nanomedicines for other indications. The adsorption of high molecular weight materials to colloidal particles is used clinically to increase antibody titer (72). A preclinical study has demonstrated that lecithin nanoparticles serve as an adjuvant to increase an immune response against protein antigens more than six times stronger than the aluminum hydroxide adjuvant (87), and Kreuter *et al*. have reported the use of polymethylmethacrylate nanoparticles as adjuvants (88).

Nanoparticles have also been associated with other hematologic safety concerns, including hemolysis and thrombogenicity. The hemolysis can be immune-mediated through nanoparticle-specific antibody or non-immunogenic through nanoparticle–erythrocyte interaction (81). Studies have shown that positive surface charge from cationic surface groups such as unprotected primary amines increases the erythrocyte damage and hemolytic potency of nanoparticles (89,90). Nanoparticles with a combination of hydrophobic and hydrophilic areas on the surfaces can act as surfactants to disrupt erythrocyte membrane (91). Hemolysis induced by nanoparticles can lead to severe side effects such as anemia. The released hemoglobin and cell debris can in turn attach to the nanoparticles, causing rapid clearance and potential immune response (92,93).

Thrombogenicity of nanoparticles is the outcome of interactions between nanoparticles and blood coagulation components and can induce blood clotting and partial or complete occlusion of blood vessels. This concern is further exacerbated by nanoparticles engineered to have longer circulation time. In the most severe form, nanoparticles can cause potentially fatal disseminated intravascular coagulation (DIC). Greish *et al*. reported that amine-terminated dendrimers triggered DIC in mice whereas carboxyl- and hydroxyl-terminated dendrimers of similar sizes were tolerated (94).

These toxicity concerns of nanoparticles present significant challenges in ensuring the safety of a nanoparticle-based medicine. As shown above, the complexity of nanoparticle systems can lead to a broad spectrum of toxicities, which are directly related to specific aspects of nanoparticle properties. A favorable safety profile would require careful adjustments of components and parameters during the development of nanoparticles. Subtle differences in composition/conformation arising from variations in manufacturing could alter the toxicities of nanoparticles, which means that a manufacturing process that is not well controlled for highly selective parameters is at risk for variations in immunogenic potential from batch to batch. In addition this has implications for potential future generic versions of nanomedicines, which may have the same components as the innovator, but could result in a different immunological profile as a result of subtle variations in composition resulting from a different manufacturing process or from inadequate control of the manufacturing process.

The testing of nanoparticle toxicity constitutes another challenge. Currently, there is no standard list of required tests. A list of *in vitro* assays can test the interaction of nanoparticles with the immune system, which include assays for hemolysis, platelet aggregation, plasma coagulation, complement activation, plasma protein binding, phagocytosis, CFU-GM, leukocyte proliferation, nitric oxide production by macrophages, and chemotaxis (81). Rodents generally are not predictive of immunological responses, while rabbits are hypersensitive to antigens. Overall, the strength of immune responses in different species follows the order of mouse < rat < dog < primate < human < rabbit. Therefore, preclinical toxicity study results, particularly those related to immunotoxicity, can not accurately predict the safety of nanomedicines in human. In clinical studies evaluating nanomedicine toxicity, special attention needs to be paid to side effects related to immune response, particularly for nanomedicines that contain a biologic component such as proteins, peptides, antibodies, and antibody fragments. It is likely that for nanomedicine products, immunological studies may have to be carried out in human clinical trials.

## REGULATORY CHALLENGES TO NANOMEDICINE DEVELOPMENT

Due to the complexity and large potential diversity of nanoparticle-based products, it may seem apparent that the regulatory pathway for nanomedicines may face several hurdles. Currently, the FDA, EMA, and other regulatory agencies examine each new nanoparticle-based drug on a product-by-product basis. There is generally a lack of standards in the examination of nanomedicines as a unique category of therapeutic agents. Recent movements towards establishing some definitions and guidelines (73,95,96) are first steps in determining if additional regulation will be applied to nanomedicines.

The complex nature of nanoparticle-based medicines with their multiple components, where more than one component can affect pharmacological behavior of the active, contrasts against standard drugs where there is usually a single active agent and the other components mostly serve as inactive formulation aids (excipients). It is reasonable to expect that nanomedicine raises complicated regulatory strategies, and processes are likely to be significantly more complex. A few fundamental and logical questions may help simplify the discussion around potential regulatory complexity of nanomedicine products while maintaining the basic principles guiding the regulatory agencies to protect public safety while providing access to new and innovative treatments:

1. Physico-chemical characteristics: what are the key characteristics of the product that are essential for its activity and safety, and are those critical characteristics of the product reproduced within acceptable pharmaceutical tolerances in manufacturing?
2. Biodistribution: are there any particular properties of the product that one would expect unusual biodistribution or more importantly cause persistence of the product in particular tissues over extended periods of time, intentionally or otherwise? If so, what are their effects?
3. Clinical: what human clinical data should be collected to evaluate potential immunological responses to the nanomedicine, whether acute or on a longer term basis upon repeated administration?
4. Definition: does the product meet the criteria of an acceptable, scientifically sound description or definition (these are still evolving) of what can be considered a nanomedicine (certain size constraints as well as unique function)?

**Fig. 5:**
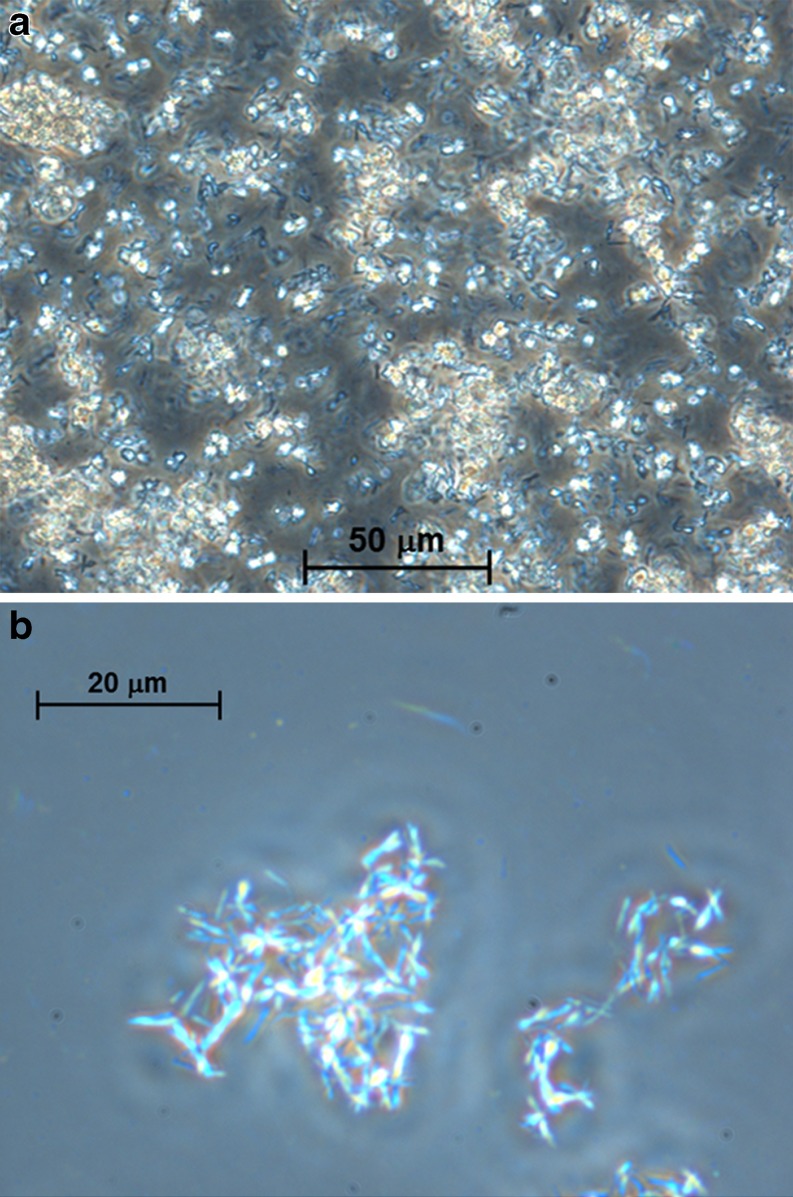
Undesirable precipitation and instability seen with an attempted albumin-paclitaxel formulation modeled as a copy for *nab*-paclitaxel. Reconstituted suspensions in saline were monitored for 24 h a 40°C. No precipitation was seen with *nab*-paclitaxel. **a** Image for the copied product at magnification of ×400 showing large visible particles; **b** higher magnification image of copied product at ×1,000 clearly shows needle-shaped crystals of paclitaxel

It should be noted that we have recently entered the era of generic nanomedicines. Both generic drug manufacturers and drug regulators will be faced with major challenges in defining what studies will be required to demonstrate that the generic nanomedicine is bioequivalent to the innovator and that the products have the same physicochemical properties and are safe and effective. For example, there have been several unsuccessful attempts in the marketplace to copy the *nab*-paclitaxel formulation. These attempted formulations which the manufacturers claimed were copies of approved *nab*-paclitaxel, when tested, failed to reproduce size distribution, stability, potency, or physicochemical characteristics of *nab*-paclitaxel, which could potentially lead to undesirable and unsafe effects. In one case, the claimed copy had high endotoxin and residual solvent levels greatly exceeding recognized safety limits. In a separate case, the attempted formulation showed substantial inter-batch variations in particle size, suggesting poor reproducibility in the manufacturing process. There was also a wide size distribution with a large portion of particles over 200 nm, resulting in significant drug loss after filtration through a 220-nm sterile filter. The reconstituted nanoparticles also displayed poor stability under accelerated conditions of 40°C and formed large precipitates and aggregates of several micrometers in size within 24 h (Fig. 5a, b), unlike *nab*-paclitaxel which was stable under these conditions. Such tests suggest that fundamental differences in the behavior of these formulations result from differences in composition and manufacturing. These examples also illustrate how generic drug manufacturers and health authorities are going to face unique challenges in the development, regulation and approval of nanomedicines that claim to be equivalent to the innovator products. These issues will likely be no less challenging than the difficulties surrounding the development and regulation of biosimilar drug products.

FDA has recently begun to consider relevant approval standards for generic copies of nanomedicines. Several liposomal type products such as those containing the drugs amphotericin and doxorubicin have recently gone off patent. Understandably, the complexity of such products should necessitate a different standard of “equivalence” testing than what is required for standard drugs. In the absence of critical information relating to composition, three dimensional configuration of components, and critical parameters that are essential for function of nanomedicine products, there may be the risk that “generic” versions are approved using the conventional chemistry, manufacturing and controls and bioequivalence standards for generic drug approvals which may result in substandard products in the marketplace. Equivalence in formulation and/or in standard blood pharmacokinetics (bioequivalence) may not adequately represent the function of the nanomedicine at the site of action, as is assumed for most standard formulations. Therefore, it is imperative that an exhaustive physicochemical understanding of complex nanomedicine products and identification of critical parameters that affect their functions be conducted early in development to lay the ground rules for potential nanomedicine generics in the future. Indeed, the FDA has recently issued a guidance for liposomal doxorubicin (Doxil) consistent with this approach (63).

In general, nanomedicines are complicated multicomponent and multifunctional drug-delivery systems. There is an urgent need for the regulatory agencies to develop a comprehensive list of tests and a streamlined approval process that covers the whole range of particle characterization, pharmacology, and toxicology issues. The overall behavior, PK, and safety profile of nanoparticles is the combined results of interplay of all nanoparticle components, parameters, and spatial composition. There is inadequate understanding of the connection between nanoparticle physicochemical properties and its clinical PK and safety, and conventional animal models may be insufficient to correctly extrapolate and predict nanoparticle biodistribution and toxicity in humans. This is especially relevant when comparing a novel nanoparticle-based drug with conventional formulations, and when evaluating a generic version of an approved nanomedicine *versus* the innovator product.

Bioequivalence of a generic and innovator nanomedicine cannot be assumed by similar results observed in general PK and toxicity studies, or by a simple comparison of the composition of the drug products. Rather, disease models should be applied when possible to reflect the pharmacology of nanomedicines at the intended target sites. Although clinical trials can assess the short-term toxicity of nanomedicines, long-term effects of their accumulation and chronic exposure may require continued monitoring over extended period of time.

Finally, many of the nanoparticle-based medicines in development consider a targeting approach to a particular receptor or protein that may be expressed, e.g., by tumors. It would be advantageous to consider a “personalized” approach to treatment and identify these subgroups of patients that are more likely to respond. Such an approach could considerably reduce the size of the required clinical trial and could be considered favorable by the regulatory agencies since a larger fraction of the patients treated under this approach would benefit from treatment. Hence, a personalized approach to nanoparticle-based medicine may not only reduce costs of running large clinical studies but could provide a speedier path to approval.

## SUMMARY AND CONCLUSIONS

In summary, a new nanoparticle-based medicine needs to successfully overcome several hurdles before it is approved for marketing. These include the development of the nanostructure with appropriate components and properties, engineering of a reproducible manufacturing process, selection of orthogonal analysis methods for adequate characterization, a favorable pharmacology and toxicity profile, and demonstration of safety and efficacy in clinical trials. While conceptually these are similar hurdles that may be faced by any new drug, the particular complexity and multicomponent nature of nanomedicines introduce large number of additional variables that may substantially increase the level of difficulty in controlling processes and predictability of behavior in a biological system. Additional regulatory and development considerations arise when generic nanomedicines are presented for health authority approval with claims of equivalence to the innovator drug.

The further development of nanomedicines will likely include a personalized medicine approach as an integral part of the clinical development strategy to identify subgroups of that particularly benefit from therapy. In addition to the many benefits of this approach and particularly true in oncology, is the reduction in size of clinical trials potentially leading to approval. Additionally, in the face of the natural biological barriers to the delivery of drugs, tissues with aberrant pathology, such as tumors, are often very efficient in harnessing active biological mechanisms for high nutrient supply and rapid growth. Elucidation of these active transport mechanisms and the ability to harness these with nanomedicines could provide a step forward in the treatment of cancer and other diseases.

Finally, health authorities that are charged with responsibility for the approval of safe and effective medicines will need to respond to the challenges posed by the emergence of products based on new technologies. Appropriate processes to develop definitions, quality standards, and requirements for development studies including clinical trials, must be in place to proactively address rapid advances in drug development.
